# MDS criteria for the diagnosis of progressive supranuclear palsy overemphasize Richardson syndrome

**DOI:** 10.1002/acn3.51065

**Published:** 2020-07-31

**Authors:** Anika Frank, Kevin Peikert, Jennifer Linn, Moritz D. Brandt, Andreas Hermann

**Affiliations:** ^1^ Department of Neurology Technische Universität Dresden Dresden Germany; ^2^ Translational Neurodegeneration Section “Albrecht‐Kossel” Department of Neurology University Medical Center Rostock University of Rostock Rostock 18147 Germany; ^3^ Department of Neuroradiology Carl Gustav Carus University Hospital Technische Universität Dresden Dresden Germany; ^4^ German Center for Neurodegenerative Diseases (DZNE) Dresden Germany; ^5^ German Center for Neurodegenerative Diseases (DZNE) Rostock/Greifswald Rostock Germany; ^6^ Center for Transdisciplinary Neurosciences Rostock (CTNR) University Medical Center Rostock University of Rostock Rostock 18147 Germany

## Abstract

MDS‐criteria for clinical diagnosis of progressive supranuclear palsy (PSP) were recently published, their usability in a classical clinical setting is yet unknown. We retrospectively applied the new criteria using PSP patients’ case files. Assignment of PSP diagnosis according to the MDS‐criteria was possible in 57/80 cases. The main difference to former specialist classification was a lower phenotype diversity and higher representation of PSP‐RS. Furthermore, we examined those patients’ brain MRIs. While neuroradiologists’ reports were suggestive of PSP only in 11/62, the analysis of a blinded rater revealed pathological midbrain‐to‐pons‐ratio in 40/62 implying this imaging feature is often missed.

## Introduction

The Movement Disorders Society (MDS) Criteria for the clinical diagnosis of progressive supranuclear palsy were recently published.^1^ While the clinical diagnostic criteria from 1996 by the National Institute of Neurological Disorders and Stroke (NINDS) have excellent specificity, their sensitivity is limited, especially early in the course of disease as well as for PSP variants other than Richardson syndrome.^2^ In correlation studies of antemortem clinical features with postmortem PSP neuropathology, only 25% of pathologically proven PSP were initially diagnosed correctly.^3^ Vertical supranuclear gaze palsy and postural instability showed the highest sensitivity for a correct, pathologically proven PSP diagnosis. Interestingly, almost all oculomotor dysfunctions had high specificity for PSP, whereas postural instability and falls only within 3 years after symptoms onset showed high specificity for PSP, however – if restricted to 3 years – on the cost of sensitivity.

The new MDS criteria aim on “improving the clinical detection of underlying PSP pathology by maintaining high diagnostic sensitivity for PSP‐RS, improving sensitivity for early and variant PSP presentations, and achieving high specificity versus alternative diagnoses”.^1^ By applying the criteria, combining different symptoms of four functional domains (“ocular motor dysfunction,” “postural stability,” “akinesia”, and “cognitive dysfunction”) being present in an individual patient result in the according PSP predominance type on a specific level of certainty (“probable,” “possible,” or “suggestive of”). Predominance types are PSP‐RS, Richardson syndrome; PSP‐PI, predominant postural instability; PSP‐OM, predominant ocular motor dysfunction; PSP‐P, predominant parkinsonism; PSP‐PGF, progressive gait freezing; PSP‐CBS, predominant corticobasal syndrome; PSP‐F, predominant frontal presentation and PSP‐SL, predominant speech/language disorder.

Only few data exist yet on the usability and practicability of the new MDS criteria in a classical movement disorder clinical setting.^4^, ^5^, ^6^, ^7^ In March 2019, Grimm et al. published a guideline for the application of the new criteria in PSP patients who show symptoms of more than one functional domain (so‐called MAX rules for multiple allocation extinction).^8^ By applying these rules the number of multiple diagnostic allocations of a single patients could be reduced from 80% to 11% of the patients or from 5.4 to 1.1 per individual.^8^

We sought to determine i) whether the new criteria including the MAX rules are suited to retrospectively assign PSP patients to the respective clinical predominance types and ii) whether and how the new classification differs compared to a movement disorders specialist diagnosis and to the NINDS‐SPSP criteria, respectively.^2^ Furthermore, we described characteristics of the new PSP phenotypes in a cohort of 80 patients (frequency, admission [mis‐]diagnosis) and analyzed the application of the midbrain‐to‐pons ratio and the Magnetic Resonance Parkinsonism Index (MRPI) as supportive diagnostic features in daily practice.

## Methods

### Patients

**Figure 1:**
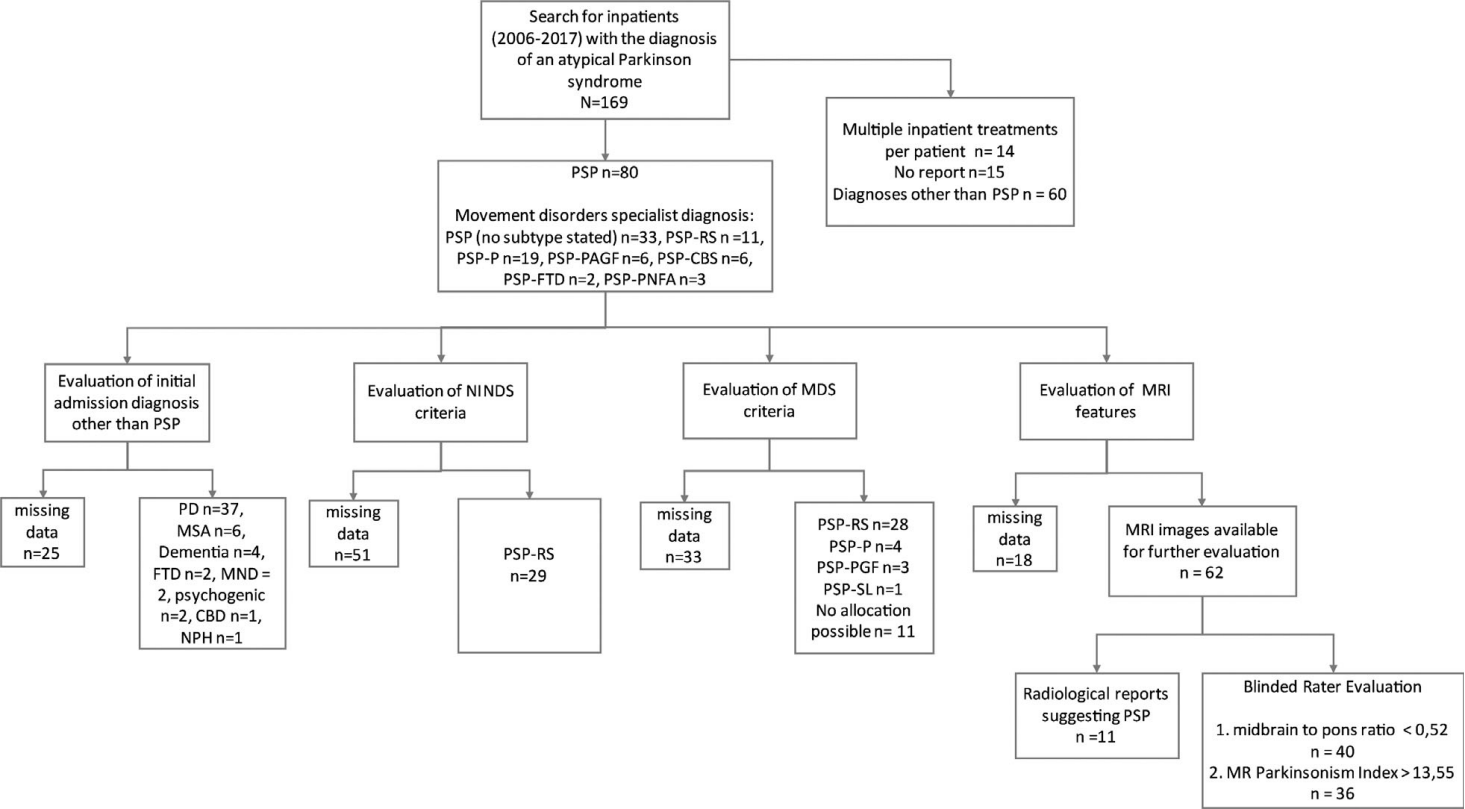
Flowchart of the study. (PSP, Progressive Supranuclear Palsy; PSP‐RS, Richardson syndrome; PSP‐PI, predominant postural instability; PSP‐OM, predominant ocular motor dysfunction; PSP‐P, predominant parkinsonism; PSP‐PGF, progressive gait freezing; PSP‐CBS, predominant corticobasal syndrome; PSP‐F, predominant frontal presentation and PSP‐SL, predominant speech/language disorder. PD, Parkinson’s disease; MSA, multiple system atrophy; FTD, frontotemporal dementia; MND, motor neuron disease; CBD, corticobasal degeneration; NPH, normal pressure hydrocephalus).

We performed a single‐center retrospective analysis of case files of movement disorders specialist care. The study was approved by the local ethics committee of the Technische Universität Dresden (EK393082019). We first screened for patients with the clinical diagnosis of PSP who were admitted to our movement disorders center at the Department of Neurology of the University Hospital Dresden from 2006 to 2017. Case files were then reviewed for the following items: Age, sex, admission diagnosis, in‐house movement disorders specialist diagnosis and PSP phenotype,^9^ crucial clinical symptoms for the application of the new MDS criteria, MRI characteristics, and neuroradiological reports. These data were then used to allocate the patients to both NINDS and MDS criteria^1^, ^2^ (Table S1). The review of case files was done by an independent movement disorder specialist neither involved in the admission nor the movement disorders specialist diagnosis that was made at our center (see flowchart, Fig. 1).

### MRI

We assessed both brain MRI reports and scans (complete image datasets available in 62/80 patients) for findings suggestive of PSP. A blinded rater (K.P.) retrospectively analyzed those brain scans using the midbrain‐to‐pons ratio in the midsagittal plane of conventional MRI.^10^, ^11^ We applied the method described by Massey et al. for this purpose; the threshold was defined as below 0.52 as suggestive of PSP.^12^ Furthermore, we applied the Magnetic resonance Parkinsonism index (MRPI), which recently was described by Nigro et al. as being more sensitive for morphological changes.^13^ The threshold for the MRPI was defined as greater than 13.55 as suggestive of PSP.^13^

### Statistical analysis

Data are shown as mean ± standard deviation (SD).

## Results

**Figure 2:**
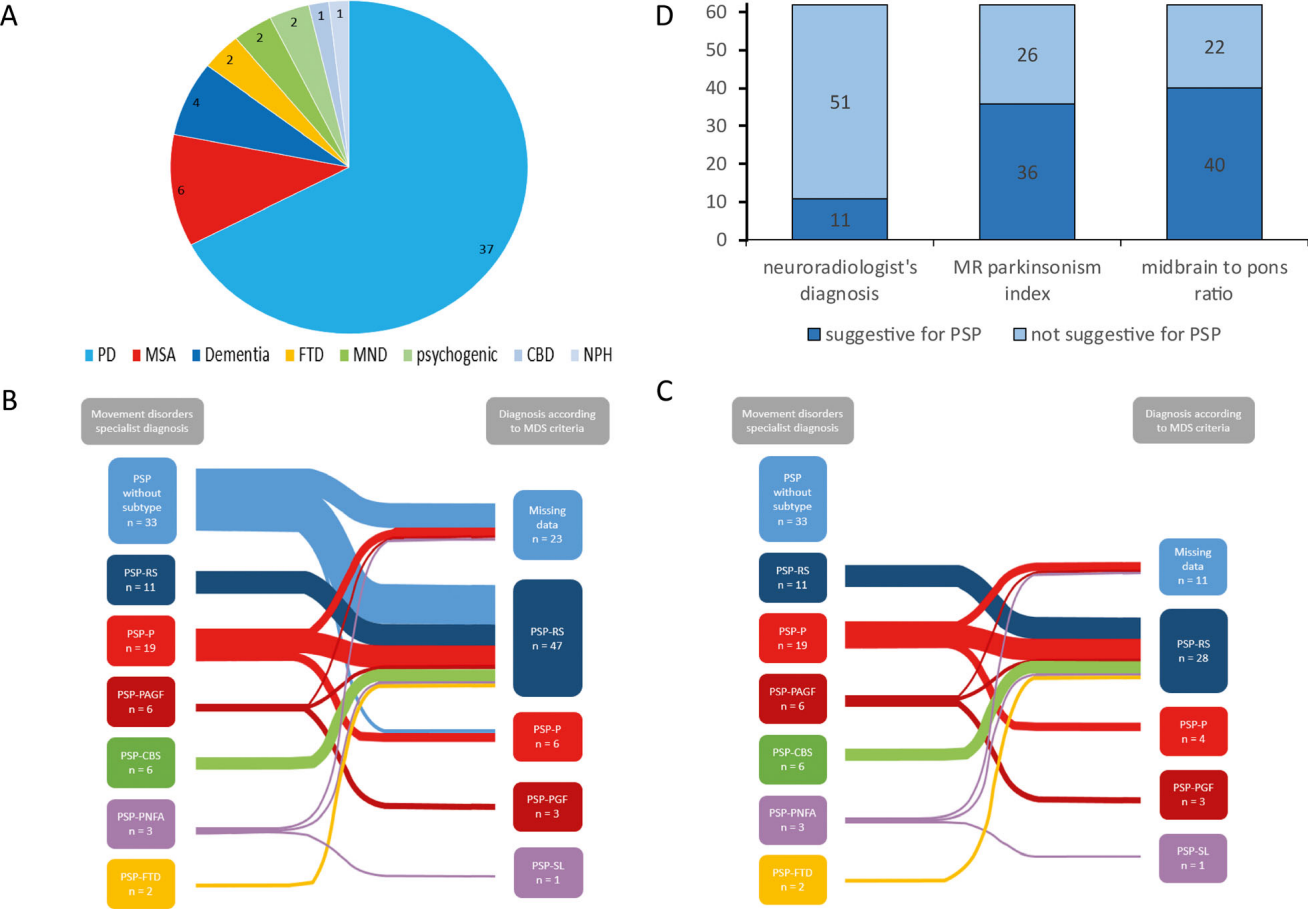
(A) Frequency of initial diagnosis before admission (*n* = 55/80) (PD, Parkinson’s disease; MSA, multiple system atrophy; FTD, frontotemporal dementia; MND, motor neuron disease; CBD, corticobasal degeneration; NPH, normal pressure hydrocephalus). (B) Frequency of PSP subgroups in our site according to movement disorder specialist opinion prior to new criteria (left column) versus new MDS‐PSP criteria (right column). (C) Frequency of PSP subgroups in our site according to movement disorder specialist opinion prior to new criteria (left column) versus new MDS‐PSP criteria (right column) when removing the group “PSP without subtype.” (D) Frequency of midbrain atrophy in MRI, either by neuroradiologist’s diagnosis (without specification how judged), by quantification of the MR parkinsonism index (MRPI) or by quantification of the midbrain to pons ratio.

We identified 80 patients with the inhouse movement disorders specialist diagnosis of PSP who were admitted to our movement disorders unit from 2006 to 2017 (male/female 52/28, age at diagnosis 70 ± 6 years, range 51–87). 55/80 patients with the movement disorder specialist diagnosis of PSP were misdiagnosed before admission, whereas Parkinson’s disease was the most frequent suspected diagnosis (Fig. 2A). The mean time from symptom onset until PSP diagnosis was 3.4 ± 2.1 years.

It was possible to assign PSP diagnosis and predominance type according to the new MDS criteria in 57/80 compared to 29/80 cases according to NINDS criteria. Lack of information about postural stability and onset of falls was the main reason for failed retrospective diagnosis and subgroup allocation in both, the MDS and the NINDS criteria. The strict time criteria for the onset of falls within the first year of disease was the main reason for the higher rate of missed retrospective diagnosis of PSP using NINDS criteria. All 29/80 patients diagnosed according to NINDS criteria were classified as PSP‐RS using the MDS criteria.

Prior to applying MAX rules, we encountered difficulties when multiple symptoms in one or even more functional domains were present in one individual patient (i.e., progressive gait freezing and symmetric parkinsonism for the akinesia domain). This was independently reproduced by Ali and colleagues.^7^ We observed multiple allocations in all our patients; without MAX rules we found 4.9 ± 1.4 diagnoses per patient. By applying MAX rules, diagnostic value of the functional domains “oculomotor dysfunction” and “postural instability” increased. This led to an increase in the diagnosis of PSP‐RS and reduced the variety of clinical phenotypes, which was most obvious for PSP‐CBS, PSP‐PGF, and PSP‐SL (Fig. 2B and C).

Finally, we investigated the frequency of midbrain atrophy as a typical MRI sign for PSP.^10^, ^11^, ^13^, ^14^ All MRI scans were evaluated by neuroradiologists. In 53/62 cases the neurologist asked specifically for radiological signs of an atypical Parkinson syndrome including a midbrain atrophy. Interestingly however, MRI scans were rated suggestive for PSP by neuroradiologists only in 11/62 cases, whereas midbrain‐to‐pons ratio was pathological in 40/62 (0.50 ± 0.09) and MRPI in 36/62 (14,7 ± 5,2) if measured by a blinded rater.^12^, ^13^ Comparing the MR images of patients fulfilling the diagnostic criteria of NINDS versus MDS showed no relevant differences concerning a pathological midbrain‐to‐pons ratio (18/23 [78.3%] from NINDS PSP‐RS group vs. 32/40 [80%] patients from MDS group of which 29/40 were classified as PSP‐RS). Those patients who were presenting with Richardson syndrome had a pathological midbrain‐to‐pons ratio in 29/37 cases (midbrain‐to‐pons ratio 0.47 ± 0.08), whereas this was the case only in 1/4 PSP‐P patients (midbrain‐to‐pons ratio 0.58 ± 0.07).

## Discussion

The MDS‐PSP criteria work well for retrospective allocation to PSP subgroups and were more suitable for identification of PSP in comparison to the former NINDS‐SPSP criteria mainly due to the wider time span of 3 years for the clinical domain of postural instability and falls. However, this also results in overemphasizing postural instability as a rather nonspecific symptom and classifying more cases as PSP‐RS (time span of 3 years: PPV for PSP 72% and specificity for PSP 81% according to Respondek and colleagues^3^) in turn reducing phenotypical diversity. This is of note since one of the main goals of the new criteria has been to enable PSP diagnosis earlier in general but specifically in rarer atypical PSP variants.^1^ Furthermore, it is crucial to differentiate between the more benign and the more progressive PSP phenotypes for prognostication for each individual patient and for further research.

A very recent study by Shoebi et al. found a similar problem displaying a poor accuracy of the MDS criteria in differentiation of PSP‐RS from PSP‐P.^6^ This study and our data are in line both giving evidence that this limits the usefulness of these criteria to differentiate between the more benign and progressive phenotypes. Thus, further refinement of the MDS criteria might be needed.

On the other hand, assigning PSP‐RS identification the highest priority might be beneficial because this subgroup correlates best with pathologically proven PSP. Both Höglinger et al., 2017 and Grimm et al., 2019 retrospectively analyzed data of autopsy‐confirmed PSP cases to establish and optimize the new MDS criteria.^1^, ^8^ In a recent antemortem clinical correlation with postmortem pathology, Respondek and colleagues revealed that vertical supranuclear gaze palsy and postural instability, both key features of the PSP‐RS phenotype, showed the highest sensitivity for an accurate PSP diagnosis.^3^ In return, some symptoms of rarer phenotypes showed high specificity for PSP but lower sensitivity (e.g., apraxia of speech or nonfluent aphasia within 3 years).^3^ On the other hand, symptoms of other rare phenotypes had low specificity for PSP neuropathology (frontal dysfunction), which makes them more likely related to different conditions (e.g., other Tauopathies). In summary, the MDS criteria seem effective in clinicopathological correlations. This is of great importance since novel causal therapeutic approaches (e.g., TAU‐antibody treatment) are arising, which need assignment to the underlying pathology.^15^

The main limitation of our study is the retrospective design. One bias of such a retrospective analysis might be the overrepresentation of the cardinal symptoms of PSP which might lead, again, to an overestimated allocation to PSP‐RS. This is specifically true when analyzing case files which include PSP diagnoses made before publication of the MDS criteria because clinicians would diagnose a PSP‐RS rather than less known PSP subtypes. Another limitation consists of the fact that our movement disorders specialist diagnosis has not been proved postmortem, which relativizes since PSP is mainly a clinical diagnosis and the majority of studies do not comprise pathological proof.

Even though midbrain atrophy might not be a biomarker for PSP neuropathology^16^ it clearly has high value to support the diagnosis, regardless of the exact methods used for quantification.^13^, ^16^ Despite this fact, our study indicates that MRI‐based quantification of midbrain atrophy is still not implemented in daily use by (neuro‐) radiologists. Thus, MRI measurements of midbrain atrophy as a supportive diagnostic feature might often be neglected, even though they are easy to administer for neurologists themselves or can be processed automatically (e.g., the MPRI).^14^, ^17^

## Conflict of Interest

AH, KP, AF, JL, and MDB declare no financial disclosure/ conflict of interest related to the manuscript.

## Author Contributions

AH designed the study. AF and KP contributed to the design, performed analysis, and evaluated specific resulting data. KP was the blinded MRI rater. AF, KP, and AH drafted and wrote the manuscript. All authors critically revised the manuscript.

## Disclosure

AF has nothing to disclose. KP has received funding from the Else Kröner‐Forschungskolleg of the Technische Universität Dresden and the MeDDrive grant of the Technische Universität Dresden. MDB has received funding from Europäischer Fonds für regionale Entwicklung (EFRE). JL has received honoria for presentations/advisory boards from Bayer Healthcare and mediaire. Andreas Hermann has received funding from the Innovationsfond des Gemeinsamen Bundesausschusses, the Helmholtz‐Association and Hermann und Lilly Schilling‐Stiftung für medizinische Forschung im Stifterverband. He has received honoraria for presentations/advisory boards from Desitin and Biogen. He has received royalties from Elsevier Press. He serves as an editorial board member of BMC neurology.

## Ethics

The study was approved by the institutional review board at the Technische Universität Dresden (EK393082019) and in accordance with the Declaration of Helsinki.

## Supporting information


**Table S1.** Application of the MDS‐PSP criteria in our single‐center cohort showing symptoms, movement disorders specialist diagnosis and possible diagnoses before application of the multiple allocation extinction rules (MAX) as well as the midbrain to pons ratio and MRPI.
