# Supplementary material for: MDS criteria for the diagnosis of progressive supranuclear palsy overemphasize Richardson syndrome
Source: Ann Clin Transl Neurol. 2020 Jul 31;7(9):1702–7. doi: 10.1002/acn3.51065 (PMC7480918; doi:10.1002/acn3.51065)
Supplement: Supplementary file 1 — Table S1. Application of the MDS‐PSP criteria in our single‐center cohort showing symptoms, movement disorders specialist diagnosis and possible diagnoses before application of the multiple allocation extinction rules (MAX) as well as the midbrain to pons ratio and MRPI. [file ACN3-7-1702-s001.pdf]

Subject	Disease Duration (years)	Ocular Motor Function (01/2/3)	Postural Instability (P1/2/3)	Akinesia (A1/2/3)	Cognitive Function (C1/2/3)	L-Dopa re-sistance	Dys-arthria	Dys-phagia	movement disorders specialist diagnosis	Diagnosis and according level of certainty applying MDS-PSP criteria before MAX rules			number of diagnoses before Max rules	NINDS PSP-RS	MRPI	Mid-brain to pons ratio
										probable	possible	suggestive of				
1	3	O2	P3	A2	0	no	yes	no	PSP	PSP-P	PSP-RS	PSP-OM	3	NA	NA	NA
2	3	O1	P1	A2	C2	yes	no	yes	PSP-RS	PSP-RS, PSP-P, PSP-F	PSP-OM	PSP-PI, PSP-P	6	yes	NA	NA
3	2	O1	P1	A2	C2	yes	no	no	PSP	PSP-RS, PSP-P, PSP-F	PSP-OM	PSP-PI, PSP-P	6	yes	NA	NA
4	2	O1	P1	A2	C2	NA	no	no	PSP	PSP-RS, PSP-P, PSP-F	PSP-OM	PSP-PI, PSP-P	6	yes	NA	NA
5	2	O1	P1	0	0	NA	yes	no	PSP	PSP-RS	PSP-OM	PSP-PI	3	yes	NA	NA
6	4		NA			NA	no	no	PSP					NA	NA	NA
7	4		NA			yes	no	no	PSP					NA	NA	NA
8	2	O1	P2	A2	C2	NA	yes	yes	PSP	PSP-RS, PSP-P, PSP-F	PSP-OM	PSP-PI, PSP-P	6	NA	NA	NA
9	2	O1	0	A2	0	yes	yes	no	PSP	PSP-P	PSP-OM		2	NA	NA	NA
10	4		NA			yes	no	no	PSP					NA	NA	NA
11	1	O1	P1	A2	0	yes	no	no	PSP	PSP-RS, PSP-P	PSP-OM	PSP-PI, PSP-P	5	yes	NA	NA
12	3	O2	P1	A3	0	yes	yes	no	PSP	PSP-RS, PSP-P		PSP-OM, PSP-PI, PSP-P	5	NA	NA	NA
13	3	0	P1	A1	0	yes	no	no	PSP-PAGF		PSP-PAGF	PSP-PI	2	NA	NA	NA
14	1	O1	P1	A2	C2	yes	no	no	PSP	PSP-RS, PSP-P, PSP-F	PSP-OM	PSP-PI, PSP-P	6	yes	NA	NA
15	2	O1	P1	A2	0	NA	yes	no	PSP	PSP-RS, PSP-P	PSP-OM	PSP-PI, PSP-P	5	NA	NA	NA
16	6	O2	P1	A2	C2	yes	yes	yes	PSP	PSP-RS, PSP-P, PSP-F		PSP-OM, PSP-PI, PSP-P	6	NA	NA	NA
17	6		NA			yes	yes	no	PSP-RS					NA	NA	NA
18	2		NA			NA	yes	no	PSP					NA	NA	NA
19	1	O1	P1	A2	C1	no	no	no	PSP-PNFA	PSP-RS, PSP-P	PSP-OM, PSP-SL	PSP-PI, PSP-P, PSP-SL	7	yes	12,08	0,45
20	1	O2	P1	A2	0	yes	yes	no	PSP-P	PSP-RS, PSP-P		PSP-OM, PSP-PI, PSP-P	5	NA	10,85	0,49
21	1	O1	P1	0	C2	yes	no	no	PSP-RS	PSP-RS, PSP-F	PSP-OM	PSP-OM, PSP-PI	5	yes	13,83	0,51
22	3	O1	P2	A2	C2	yes	yes	yes	PSP-P	PSP-RS, PSP-P, PSP-F	PSP-OM	PSP-PI; PSP-P	6	NA	13,69	0,49
23	10	O1	0	A2	0	yes	yes	yes	PSP-P	PSP-P	PSP-OM		2	NA	5,88	0,61
24	3	O1	P1	A2	C3	yes	yes	yes	PSP-CBS	PSP-RS, PSP-P	PSP-OM, PSP-CBS	PSP-PI, PSP-P, PSP-CBS	7	NA	12,56	0,44
25	2	O2	P1	0	C2	yes	yes	no	PSP-FTD	PSP-RS, PSP-F		PSP-OM, PSP-PI	4	NA	13,73	0,46
26	2	O1	P1	A3	C3	yes	yes	no	PSP-CBS	PSP-RS, PSP-P	PSP-OM, PSP-CBS	PSP-PI, PSP-P	6	yes	17,05	0,64
27	4	O2	0	A3	0	yes	yes	no	PSP-P	PSP-P		PSP-OM, PSP-P	3	NA	9,38	0,60

28	2	O1	P1	0	0	yes	no	no	PSP-RS	PSP-RS	PSP-OM,	PSP-PI	3	yes	14,26	0,48
29	1	O1	P2	A2	C2	yes	yes	no	PSP	PSP-RS, PSP-P, PSP-F	PSP-OM	PSP-PI, PSP-P	6	NA	16,12	0,46
30	NA		NA			yes	yes	yes	PSP					NA	10,09	0,60
31	1	O1	P1	A3	C2	yes	yes	yes	PSP	PSP-RS, PSP-P, PSP-F	PSP-OM	PSP-PI, PSP-P	6	yes	18,58	0,47
32	3	O2	0	A3	0	yes	yes	no	PSP-P	PSP-P		PSP-OM, PSP-P	3	NA	8,67	0,66
33	3		NA			yes	yes	yes	PSP					NA	12,08	0,53
34	11	O1	P3	A2	0	yes	yes	no	PSP-P	PSP-P	PSP-OM	PSP-P	3	NA	10,31	0,46
35	4	O1	P1	0	C1	yes	yes	no	PSP-FTD	PSP-RS	PSP-OM, PSP-SL	PSP-PI, PSP-SL	5	NA	13,96	0,44
36	NA		NA			yes	yes	yes	PSP					NA	16,20	0,45
37	1	O1	P1	A3	0	yes	no	no	PSP-P	PSP-RS, PSP-P	PSP-OM	PSP-PI, PSP-P	5	yes	18,96	0,50
38	NA		NA			yes	no	no	PSP					NA	8,86	0,69
39	4	O1	P2	A2	C2	yes	no	no	PSP	PSP-RS, PSP-P, PSP-F	PSP-OM	PSP-PI, PSP-P	6	NA	14,62	0,55
40	NA		NA			yes	yes	no	PSP-RS					NA	19,11	0,49
41	1	O1	P1	0	0	yes	no	no	PSP-RS	PSP-RS	PSP-OM,	PSP-PI	3	yes	17,39	0,42
42	3	O1	P2	A2	C3	yes	yes	no	PSP-CBS	PSP-RS, PSP-P	PSP-OM, PSP-CBS	PSP-PI, PSP-P, PSP-CBS	7	NA	7,81	0,66
43	7		NA			yes	yes	no	PSP-P					yes	9,21	0,55
44	4	O2	P1	0	C1	yes	yes	no	PSP-P	PSP-RS	PSP-SL	PSP-OM, PSP-PI, PSP-SL	5	yes	19,27	0,36
45	5		NA			yes	yes	no	PSP-PAGF					NA	17,41	0,51
46	5		NA			yes	yes	no	PSP					NA	10,39	0,62
47	6	O1	P1	A2	0	yes	yes	yes	PSP-RS	PSP-RS, PSP-P	PSP-OM	PSP-PI, PSP-P	5	NA	17,94	0,31
48	2	O1	P1	A2	0	yes	no	no	PSP	PSP-RS, PSP-P	PSP-OM	PSP-PI, PSP-P	5	yes	15,12	0,54
49	1	O1	P1	A2	0	yes	no	no	PSP-Rs	PSP-RS, PSP-P	PSP-OM	PSP-PI, PSP-P	5	yes	14,69	0,47
50	3	O1	P1	A2	C2	yes	yes	no	PSP-RS	PSP-RS, PSP-P, PSP-F	PSP-OM	PSP-PI, PSP-P	6	yes	11,12	0,42
51	4	O2	P1	A2	C3	yes	no	no	PSP-CBS	PSP-RS, PSP-P	PSP-CBS	PSP-OM, PSP-PI, PSP-P, PSP-CBS	7	yes	14,67	0,41
52	1	O1	P1	A2	0	yes	yes	no	PSP	PSP-RS, PSP-P	PSP-OM	PSP-PI, PSP-P	5	yes	15,43	0,51
53	3	O2	P1	A3	0	no	yes	no	PSP-P	PSP-RS, PSP-P		PSP-OM, PSP-PI, PSP-P	5	NA	8,94	0,71
54	4	O1	P1	A2	C2	yes	yes	no	PSP-P	PSP-RS, PSP-P, PSP-F	PSP-OM	PSP-PI, PSP-P	6	yes	18,86	0,41
55	3	O1	P1	A2	0	yes	yes	no	PSP-P	PSP-RS, PSP-P	PSP-OM	PSP-PI, PSP-P	5	yes	12,49	0,53
56	2		NA			yes	yes	no	PSP-P					NA	12,06	0,57
57	2	O1	P1	A2	0	yes	yes	no	PSP	PSP-RS, PSP-P	PSP-OM	PSP-PI, PSP-P	5	yes	20,94	0,35
58	2	O2	P1	A2	0	yes	no	no	PSP-P	PSP-RS, PSP-P		PSP-OM, PSP-PI, PSP-P	5	NA	13,87	0,49

59	6	O1	P1	A1	0	yes	yes	no	PSP-PAGF	PSP-RS, PSP-PAGF	PSP-OM, PSP-PAGF	PSP-PI	5	yes	8,99	0,53
60	4		NA			NA	yes	yes	PSP-PNFA					NA	13,44	0,56
61	4	O1	0	0	C1	NA	yes	no	PSP-PNFA		PSP-OM, PSP-SL	PSP-SL	3	NA	18,23	0,45
62	1	O1	P1	A2	C2	yes	yes	no	PSP-RS	PSP-RS, PSP-P, PSP-F	PSP-OM	PSP-PI, PSP-P	6	yes	22,27	0,40
63	4	O1	P1	A1	C2	yes	yes	no	PSP-PAGF	PSP-RS, PSP-PAGF, PSP-F	PSP-OM, PSP-PAGF	PSP-PI	6	yes	9,82	0,52
64	5		NA			NA	yes	no	PSP					NA	15,27	0,48
65	4		NA			yes	no	no	PSP-RS					NA	10,56	0,59
66	5		NA			yes	yes	no	PSP-CBS					nen	31,69	0,40
67	3	O2	P1	A2	C2	yes	yes	yes	PSP-P	PSP-RS, PSP-P, PSP-F		PSP-OM, PSP-PI, PSP-P	6	NA	14,79	0,55
68	5		NA			yes	no	no	PSP					NA	17,66	0,50
69	1	O1	P1	A2	0	NA	yes	no	PSP	PSP-RS, PSP-P	PSP-OM	PSP-PI, PSP-P	5	yes	18,47	0,40
70	1	O1	P1	A2	0	yes	yes	no	PSP	PSP-RS, PSP-P	PSP-OM	PSP-PI, PSP-P	5	yes	27,55	0,46
71	5		NA			NA	no	no	PSP					NA	6,75	0,75
72	5	O1	P1	A3	0	yes	no	no	PSP-P	PSP-RS, PSP-P	PSP-OM	PSP-PI, PSP-P	5	NA	22,91	0,47
73	1	0	P1	A1	0	yes	yes	no	PSP-PAGF		PSP-PAGF	PSP-PI	2	NA	14,68	0,52
74	3	O2	P1	A1	C3	NA	no	no	PSP-P	PSP-RS, PSP-PAGF	PSP-PAGF	PSP-OM, PSP-PI	4	NA	9,58	0,52
75	8	O1	P1	A2	C3	NA	yes	yes	PSP-CBS	PSP-RS, PSP-P	PSP-OM, PSP-CBS	PSP-PI, PSP-P, PSP-CBS	7	NA	27,97	0,36
76	1	O2	P1	0	0	NA	no	no	PSP	PSP-RS		PSP-OM, PSP-PI	3	yes	11,45	0,47
77	5		NA			yes	yes	yes	PSP					NA	8,59	0,61
78	5	O2	P3	A1	0	yes	no	no	PSP-PAGF	PSP-PAGF	PSP-RS, PSP-PAGF	PSP-OM	4	NA	23,18	0,40
79	4		NA			yes	yes	no	PSP-P					NA	14,34	0,42
80	10		NA			yes	yes	no	PSP-P					NA	15,99	0,48

Table 1: Application of the MDS-PSP criteria in our single center cohort showing symptoms, movement disorders specialist diagnosis and possible diagnoses before application of the multiple allocation extinction rules (MAX) as well as the measured midbrain to pons ratio and MRPI.
